# Diagnostic Value of Systemic Inflammatory Indices in Myocardial İnfarction With Non-Obstructive Coronary Arteries: Comparison With Obstructive Myocardial Infarction and Non-Critical Coronary Artery Disease

**DOI:** 10.14740/cr2290

**Published:** 2026-08-31

**Authors:** Abdullah Emre Bektas, Cagri Zorlu, Atac Celik, Ahmet Avci, Kayihan Karaman, Metin Karayakali, Gulsen Genc Tapar, Sefa Erdi Omur, Osman Demir

**Affiliations:** a Department of Cardiology, Kastamonu Training and Research Hospital, Kastamonu, Turkey; b Department of Cardiology, Tokat Gaziosmanpasa University Faculty of Medicine, Tokat, Turkey; c Department of Cardiology, Kastamonu University Faculty of Medicine, Kastamonu, Turkey; d Department of Biostatistics, Tokat Gaziosmanpasa University, Tokat, Turkey

**Keywords:** MINOCA, Myocardial infarction, Systemic immune-inflammation index, Neutrophil-to-lymphocyte ratio, İnflammation, Echocardiography

## Abstract

**Background:**

Myocardial infarction with non-obstructive coronary arteries (MINOCA) represents a heterogeneous clinical entity with diverse underlying mechanisms, and its diagnostic evaluation remains challenging in routine clinical practice despite advances in multimodality imaging. Systemic inflammatory indices derived from routine laboratory parameters have recently gained attention as potential markers reflecting inflammatory burden in cardiovascular diseases. This study aimed to evaluate the potential diagnostic utility of systemic inflammatory indices in patients with MINOCA and to compare these indices across patients with obstructive myocardial infarction and non-critical coronary artery disease.

**Methods:**

In this retrospective single-center observational study, a total of 2,050 consecutive patients who underwent coronary angiography between January 1, 2023, and January 1, 2024 were initially screened. After applying predefined exclusion criteria, including renal failure, pulmonary embolism, cerebrovascular events, arrhythmias, severe valvular heart disease, severe anemia, and incomplete clinical data, 496 patients were eligible for the final analysis. Patients were classified into three groups according to coronary angiographic findings and troponin levels: MINOCA (n = 67), obstructive myocardial infarction (n = 222), and non-critical coronary artery disease (n = 207). Demographic characteristics, laboratory parameters, echocardiographic findings, and systemic inflammatory indices including neutrophil-to-lymphocyte ratio (NLR), platelet-to-lymphocyte ratio (PLR), monocyte-to-lymphocyte ratio (MLR), systemic immune-inflammation index (SII), and C-reactive protein-to-albumin ratio (CAR) were analyzed. Multivariable logistic regression analysis together with receiver operating characteristic (ROC) curve analysis was performed to determine independent predictors associated with MINOCA.

**Results:**

Patients with obstructive myocardial infarction were older and had a higher prevalence of diabetes mellitus and hypertension compared with the MINOCA and non-critical coronary artery disease groups (P < 0.001). Systemic inflammatory indices including neutrophil count, SII, NLR, PLR, MLR, C-reactive protein (CRP), and CAR were significantly higher in the obstructive myocardial infarction group, whereas intermediate levels were observed in the MINOCA group (P < 0.001). Left ventricular ejection fraction (LVEF) was significantly lower in the obstructive myocardial infarction group compared with the MINOCA and non-critical coronary artery disease groups (P < 0.001). In multivariate logistic regression analysis, LVEF was identified as an independent predictor of MINOCA when compared with obstructive myocardial infarction, whereas SII remained an independent predictor in comparisons between MINOCA and non-critical coronary artery disease groups. Receiver operating characteristic (ROC) curve analyses demonstrated that LVEF showed the highest diagnostic performance among the evaluated parameters.

**Conclusions:**

Systemic inflammatory indices differ significantly among patients with MINOCA, obstructive myocardial infarction, and non-critical coronary artery disease. Easily accessible inflammatory markers together with echocardiographic parameters such as LVEF may provide additional diagnostic value in the clinical evaluation of patients with suspected MINOCA.

## Introduction

Myocardial infarction remains one of the leading causes of morbidity and mortality worldwide despite substantial advances in early reperfusion strategies and contemporary pharmacological therapies [1, 2]. Inflammation plays a central role in the pathophysiology of acute coronary syndromes by contributing to atherosclerotic plaque formation, progression, and plaque rupture [3]. In addition to plaque destabilization, inflammatory pathways also contribute to endothelial dysfunction and thrombus formation during acute coronary syndromes [4]. In recent years, several systemic inflammatory indices derived from routine hematological parameters, including neutrophil-to-lymphocyte ratio (NLR), platelet-to-lymphocyte ratio (PLR), monocyte-to-lymphocyte ratio (MLR), C-reactive protein-to-albumin ratio (CAR), and systemic immune-inflammation index (SII), have been investigated as potential markers reflecting inflammatory burden in cardiovascular diseases [5]. Among these indices, the SII has emerged as a promising marker associated with disease severity and prognosis in patients with cardiovascular diseases, particularly acute coronary syndromes [6].

Myocardial infarction with non-obstructive coronary arteries (MINOCA) represents a heterogeneous clinical entity characterized by evidence of myocardial infarction in the absence of significant obstructive coronary artery disease on angiography [7]. Contemporary diagnostic strategies recommend the use of multimodality imaging, including cardiac magnetic resonance and intracoronary imaging techniques, to identify the underlying mechanism in patients with MINOCA [8]. MINOCA accounts for approximately 5–10% of all myocardial infarction cases and includes various underlying mechanisms such as plaque disruption, coronary vasospasm, microvascular dysfunction, myocarditis, and Takotsubo cardiomyopathy [9, 10]. Because of its heterogeneous pathophysiology and diagnostic complexity, identifying reliable biomarkers that may assist in differentiating MINOCA from obstructive myocardial infarction remains clinically important [7].

Although several studies have evaluated the association between systemic inflammatory markers and obstructive myocardial infarction, data regarding their role in patients with MINOCA remain limited [7, 11]. In particular, comparative evaluation of multiple systemic inflammatory indices across different coronary angiographic presentations has not been sufficiently investigated [12].

Accordingly, this study was designed to comprehensively evaluate the diagnostic utility of multiple systemic inflammatory indices (NLR, PLR, MLR, SII, and CAR) in patients with MINOCA and to compare these parameters with those observed in patients with obstructive myocardial infarction and non-critical coronary artery disease.

## Materials and Methods

**Figure 1:**
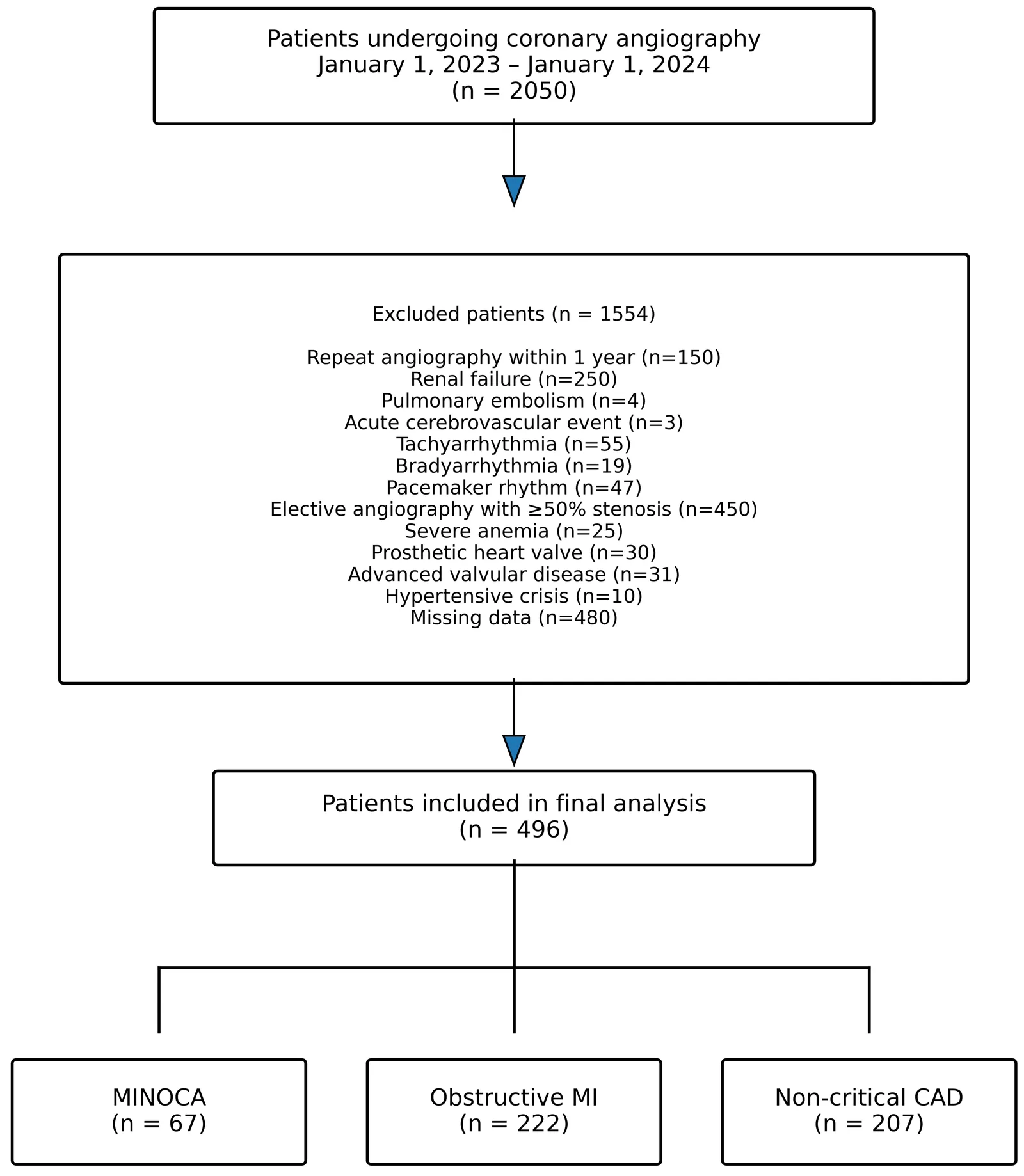
Flow diagram of patient selection and study group allocation. MINOCA: myocardial infarction with non-obstructive coronary arteries; MI: myocardial infarction; CAD: coronary artery disease.

This retrospective single-center study initially screened 2,050 consecutive patients who underwent coronary angiography at Tokat Gaziosmanpasa University Faculty of Medicine between January 1, 2023, and January 1, 2024. Patients meeting predefined exclusion criteria were excluded from the study population. In addition, patients with normal cardiac troponin levels who underwent elective coronary angiography and were found to have ≥ 50% coronary artery stenosis were excluded. After applying all inclusion and exclusion criteria, 496 patients were included in the final analysis and classified into three groups according to coronary angiographic findings and cardiac troponin levels: the MINOCA group (n = 67), the obstructive myocardial infarction group (n = 222), and the non-critical coronary artery disease group (n = 207). The patient selection process is summarized in Figure 1.

MINOCA diagnosis was established according to the 2019 American Heart Association (AHA) Scientific Statement criteria, including clinical evidence of myocardial infarction, absence of obstructive coronary artery disease (no coronary stenosis ≥ 50%), and no overt alternative diagnosis for the acute presentation [7].

Patients with elevated cardiac troponin levels and absence of ≥ 50% coronary artery stenosis on coronary angiography were classified as the MINOCA group. Patients with elevated cardiac troponin levels and ≥ 50% stenosis in at least one epicardial coronary artery were included in the obstructive myocardial infarction group. Patients with normal cardiac troponin levels and < 50% stenosis on coronary angiography who underwent elective angiography were classified as the non-critical coronary artery disease group.

Patients with active infection, chronic inflammatory disease, malignancy, hematological disorders, or those receiving immunosuppressive therapy were excluded from the study. The patient selection process, including reasons for exclusion and final study group allocation, is summarized in Figure 1.

Demographic characteristics, cardiovascular risk factors, laboratory parameters, echocardiographic findings, and systemic inflammatory indices including NLR, PLR, MLR, SII, and CAR were recorded from hospital electronic medical records.

Transthoracic echocardiographic examinations were performed in accordance with current guideline recommendations, and left ventricular ejection fraction (LVEF) and systolic pulmonary artery pressure (SPAP) values were recorded.

All statistical analyses were conducted using IBM SPSS Statistics for Windows (version 26.0, IBM Corp., Armonk, NY, USA). Continuous variables were expressed as mean ± standard deviation, and categorical variables were presented as numbers and percentages. Group comparisons were performed using appropriate parametric or non-parametric statistical tests according to data distribution. Logistic regression analyses were performed to identify independent predictors of MINOCA variables with a P value < 0.10 in univariate analysis, together with clinically relevant parameters including age, sex, diabetes mellitus, hypertension and LVEF were entered into the multivariable logistic regression model. Receiver operating characteristic (ROC) curve analyses were used to determine optimal cut-off values for significant parameters. A P value < 0.05 was considered statistically significant.

The study protocol received approval from the Clinical Research Ethics Committee of Tokat Gaziosmanpasa University Faculty of Medicine (approval number: 22-KAEK-271, December 8, 2022) and the study procedures complied with the principles outlined in the Declaration of Helsinki.

## Results

A total of 496 patients were included in the study: 67 patients in the MINOCA group, 222 patients in the obstructive myocardial infarction group, and 207 patients in the non-critical coronary artery disease group. The mean age of the overall study population was 61.87 ± 12.22 years. Patients in the obstructive myocardial infarction group were significantly older than those in both the MINOCA and non-critical coronary artery disease groups (66.32 ± 10.93 vs. 56.75±16.09 and 58.76 ± 10.39 years, respectively; P < 0.001).

Male sex was significantly more frequent in the obstructive myocardial infarction group compared with both the MINOCA and non-critical coronary artery disease groups (70.7%, P < 0.001). Similarly, diabetes mellitus and hypertension were significantly more prevalent in the obstructive myocardial infarction group than in the MINOCA and non-critical coronary artery disease groups (48.2% and 48.2%, respectively; both P < 0.001). There was no statistically significant difference between the groups in terms of smoking status (P = 0.631). Baseline demographic characteristics of the study population are presented in Table 1.

**Table 1:** Baseline Demographic and Clinical Characteristics of the Study Population

Variable	MINOCA (n = 67)	Obstructive MI (n = 222)	Non-critical CAD (n = 207)	P value
Age (years)	56.75 ± 16.09	66.32 ± 10.93	58.76 ± 10.39	< 0.001
Male sex, n (%)	37 (55.2)	157 (70.7)	111 (53.6)	< 0.001
Diabetes mellitus, n (%)	18 (26.9)	107 (48.2)	64 (30.9)	< 0.001
Hypertension, n (%)	21 (31.3)	107 (48.2)	61 (29.5)	< 0.001
Smoking, n (%)	27 (40.3)	79 (35.6)	75 (36.2)	0.631

Values are expressed as mean ± standard deviation or number (percentage). CAD: coronary artery disease; MI: myocardial infarction; MINOCA: myocardial infarction with non-obstructive coronary arteries.

Echocardiographic parameters including LVEF and SPAP were evaluated in all groups. The mean LVEF value was significantly lower in the obstructive myocardial infarction group than in both the MINOCA and non-critical coronary artery disease groups (48.69 ± 9.06 vs. 53.81 ± 8.71 and 58.77 ± 5.17, respectively; P < 0.001). The highest LVEF values were observed in the non-critical coronary artery disease group.

The mean SPAP value was significantly lower in the non-critical coronary artery disease group compared with the MINOCA and obstructive myocardial infarction groups (28.55 ± 6.30 vs. 31.27 ± 8.81 and 31.89 ± 6.75, respectively; P < 0.001). Echocardiographic findings are presented in Table 2.

**Table 2:** Echocardiographic Parameters of the Study Population

Variable	MINOCA (n = 67)	Obstructive MI (n = 222)	Non-critical CAD (n = 207)	P value
LVEF (%)	53.81 ± 8.71	48.69 ± 9.06	58.77 ± 5.17	< 0.001
SPAP (mm Hg)	31.27 ± 8.81	31.89 ± 6.75	28.55 ± 6.30	< 0.001

Values are expressed as mean ± standard deviation. LVEF: left ventricular ejection fraction; SPAP: systolic pulmonary artery pressure; CAD: coronary artery disease; MI: myocardial infarction; MINOCA: myocardial infarction with non-obstructive coronary arteries.

Laboratory parameters were compared among the three study groups. Serum glucose and creatinine levels were significantly higher in the obstructive myocardial infarction group compared with the MINOCA and non-critical coronary artery disease groups (both P < 0.001). Similarly, low-density lipoprotein (LDL) cholesterol levels were higher in the obstructive myocardial infarction group than in the other groups (P = 0.004).

Serum albumin levels were significantly lower in the obstructive myocardial infarction group than in the MINOCA and non-critical coronary artery disease groups (P < 0.001). Hemoglobin levels also differed significantly among the groups, with lower values observed in the obstructive myocardial infarction group (P = 0.005). In addition, white blood cell counts were significantly higher in the obstructive myocardial infarction group compared with the other groups (P < 0.001).

Systemic inflammatory indices were significantly different among the three study groups. Neutrophil counts were highest in the obstructive myocardial infarction group, followed by the MINOCA group and the non-critical coronary artery disease group (6.65 ± 2.46 vs. 5.72 ± 2.14 vs. 4.57 ± 1.48, respectively; P < 0.001). Similarly, lymphocyte counts were significantly lower in the obstructive myocardial infarction group compared with the other groups (P < 0.001).

SII values were significantly higher in the obstructive myocardial infarction group compared with the MINOCA and non-critical coronary artery disease groups (931.90 ± 739.43 vs. 756.12 ± 660.39 vs. 505.64 ± 245.85, respectively; P < 0.001). Likewise, NLR values were highest in the obstructive myocardial infarction group, followed by the MINOCA group and the non-critical coronary artery disease group (4.01 ± 2.87 vs. 3.17 ± 2.54 vs. 2.17 ± 1.06, respectively; P < 0.001).

PLR and MLR values also differed significantly among the groups. Both parameters were higher in the obstructive myocardial infarction group compared with the other groups (P < 0.001 for both comparisons). In addition, C-reactive protein (CRP) and CAR values were significantly elevated in the obstructive myocardial infarction group compared with the MINOCA and non-critical coronary artery disease groups (both P < 0.001). Laboratory parameters and systemic inflammatory indices are summarized in Table 3.

**Table 3:** Laboratory Parameters and Systemic Inflammatory Indices of the Study Population

Variable	MINOCA (n = 67)	Obstructive MI (n = 222)	Non-critical CAD (n = 207)	P value
Neutrophil	5.7 ± 2.14	6.65 ± 2.46	4.57 ± 1.48	< 0.001
Lymphocyte	2.26 ± 0.92	2.08 ± 0.91	2.33 ± 0.77	0.009
Platelet	242.27 ± 71.13	231.92 ± 65.12	238.05 ± 60.63	0.418
Monocyte	0.69 ± 0.25	0.72 ± 0.27	0.64 ± 0.20	0.005
Hemoglobin	13.14 ± 1.72	12.99 ± 1.97	13.39 ± 1.79	0.080
WBC	8.88 ± 2.45	9.61 ± 2.65	7.80 ± 1.96	< 0.001
Creatinine	0.91 ± 0.23	1.07 ± 0.32	0.88 ± 0.20	< 0.001
Glucose	116.53 ± 42.53	156.61 ± 73.10	115.86 ± 39.30	< 0.001
Albumin	4.01 ± 0.35	3.97 ± 0.41	4.30 ± 0.37	< 0.001
LDL	111.91 ± 35.31	123.99 ± 38.08	113.46 ± 35.58	0.004
SII	756.12 ± 660.39	931.90 ± 739.43	505.64 ± 245.85	< 0.001
NLR	3.17 ± 2.54	4.01 ± 2.87	2.17 ± 1.06	< 0.001
PLR	125.89 ± 67.75	133.00 ± 69.80	111.69 ± 41.20	0.001
MLR	0.35 ± 0.24	0.39 ± 0.22	0.30 ± 0.11	< 0.001
CRP	10.11 ± 15.17	17.54 ± 26.98	5.25 ± 8.24	< 0.001
CAR	2.52 ± 3.74	4.70 ± 7.66	1.26 ± 2.00	< 0.001
ProBNP	1,201.55 ± 1,904.01	2,940.03 ± 4,953.32	164.78 ± 268.23	< 0.001

Values are expressed as mean ± standard deviation. WBC: white blood cell count; LDL: low-density lipoprotein; SII: systemic immune-inflammation index; NLR: neutrophil-to-lymphocyte ratio; PLR: platelet-to-lymphocyte ratio; MLR: monocyte-to-lymphocyte ratio; CRP: C-reactive protein; CAR: CRP-to-albumin ratio; CAD: coronary artery disease; MI: myocardial infarction; MINOCA: myocardial infarction with non-obstructive coronary arteries; proBNP: pro–B-type natriuretic peptide.

Logistic regression analyses were performed to identify independent predictors of MINOCA. In univariate logistic regression analysis comparing the MINOCA and obstructive myocardial infarction groups, neutrophil count, LVEF, SII, and NLR were found to be significantly associated with the diagnosis of MINOCA (all P < 0.05). However, in multivariate logistic regression analysis, only LVEF remained an independent predictor of MINOCA compared with obstructive myocardial infarction (odds ratio (OR), 0.936; 95% CI, 0.901–0.972; P = 0.001).

Similarly, in the comparison between the MINOCA and non-critical coronary artery disease groups, neutrophil count, SII, and NLR were identified as significant predictors in univariate analysis (all P < 0.05). Among these parameters, SII remained an independent predictor of MINOCA in multivariate logistic regression analysis (P = 0.001).

**Figure 2:**
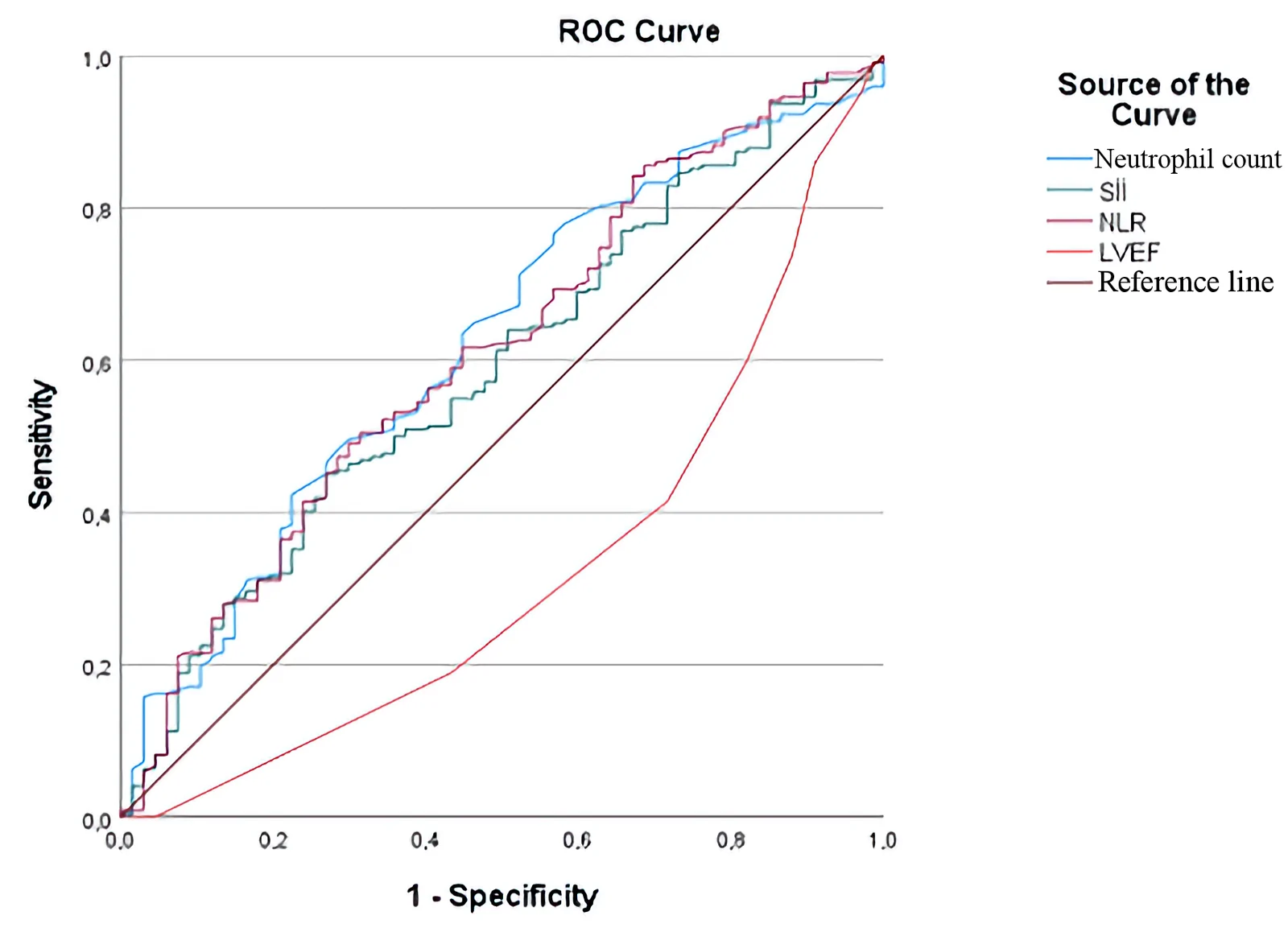
Receiver operating characteristic (ROC) curve analysis of neutrophil count, systemic immune-inflammation index (SII), neutrophil-to-lymphocyte ratio (NLR), and left ventricular ejection fraction (LVEF) for differentiation between MINOCA and obstructive myocardial infarction (group 1 vs group 2). MINOCA: myocardial infarction with non-obstructive coronary arteries.

**Figure 3:**
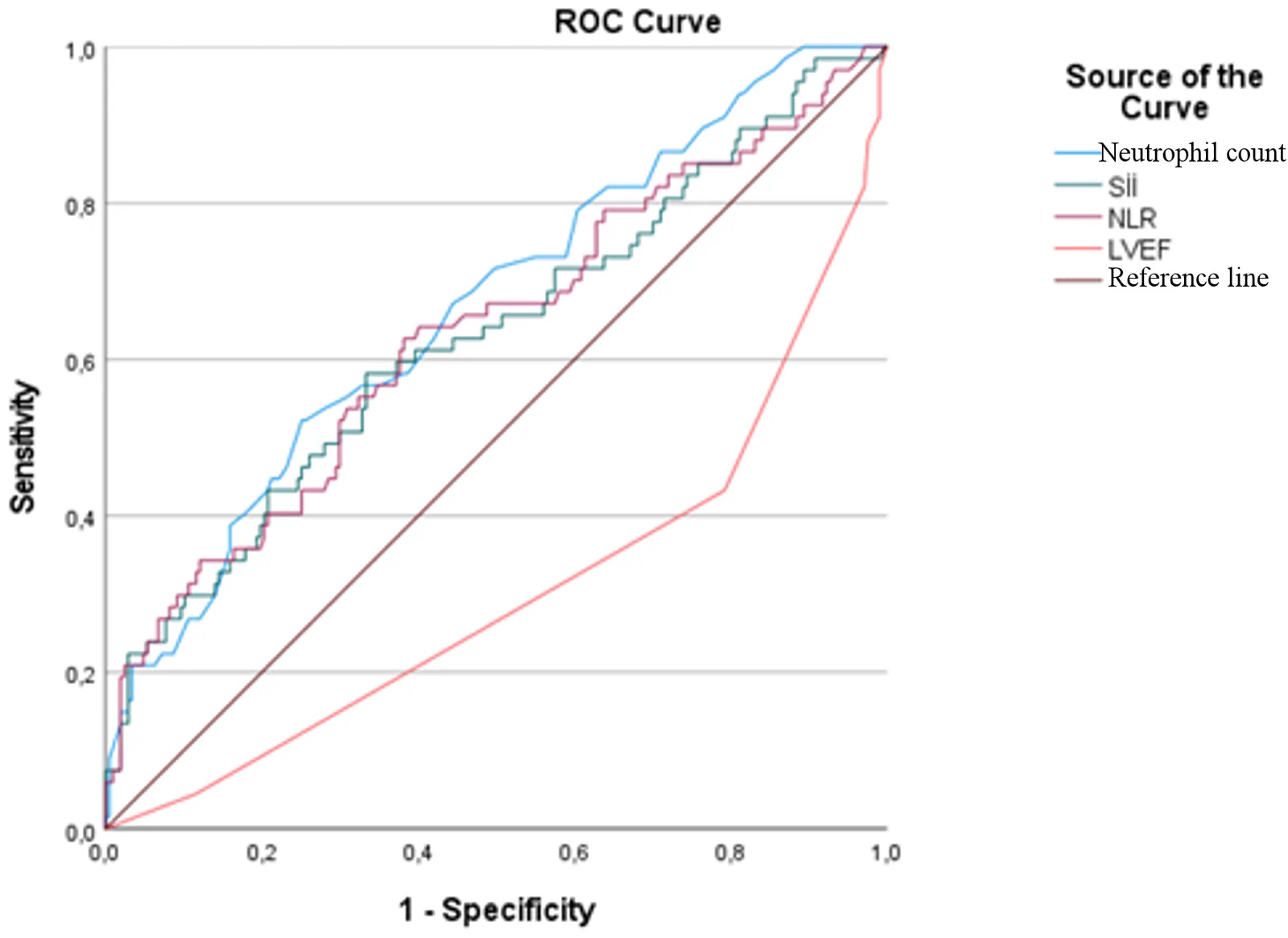
Receiver operating characteristic (ROC) curve analysis of neutrophil count, systemic immune-inflammation index (SII), neutrophil-to-lymphocyte ratio (NLR), and left ventricular ejection fraction (LVEF) for differentiation between MINOCA and non-critical coronary artery disease (group 1 vs group 3). MINOCA: myocardial infarction with non-obstructive coronary arteries.

ROC curve analyses were performed to evaluate the diagnostic performance of inflammatory parameters in distinguishing MINOCA from obstructive myocardial infarction. The area under the curve (AUC) for neutrophil count was 0.622, with an optimal cut-off value of ≥ 6.7 providing 42.3% sensitivity and 77.6% specificity (P = 0.002). The AUC value for SII was 0.589, and a cut-off value of ≥ 796.44 yielded 45.1% sensitivity and 73.1% specificity (P = 0.022). Similarly, the AUC for NLR was 0.611, with a cut-off value of ≥ 3.23 providing 49.1% sensitivity and 70.2% specificity (P = 0.004). LVEF demonstrated the highest diagnostic performance among these parameters, with an AUC of 0.678 and a cut-off value of ≤ 50 showing 58.6% sensitivity and 71.6% specificity (P < 0.001). In the comparison between MINOCA and obstructive myocardial infarction (group 1 vs group 2), neutrophil count, SII, NLR, and LVEF demonstrated significant discriminative ability (Fig. 2, Table 4). Similarly, in the comparison between MINOCA and non-critical coronary artery disease (group 1 vs group 3), these parameters also showed significant diagnostic performance (Fig. 3, Table 5).

**Table 4:** ROC Analysis Results for Differentiation of MINOCA and Obstructive Myocardial Infarction

Variable	Cut-off	AUC	Sensitivity	Specificity	PPV	NPV	P value
Neutrophil	≥ 6.7	0.622	0.423	0.776	0.862	0.289	0.002
SII	≥ 796.44	0.589	0.451	0.731	0.848	0.287	0.022
NLR	≥ 3.23	0.611	0.491	0.702	0.845	0.294	0.004
LVEF	≤ 50	0.678	0.586	0.716	0.873	0.343	< 0.001

AUC: area under the curve; PPV: positive predictive value; NPV: negative predictive value; SII: systemic immune-inflammation index; NLR: neutrophil-to-lymphocyte ratio; LVEF: left ventricular ejection fraction; ROC: receiver operating characteristic; MINOCA: myocardial infarction with non-obstructive coronary arteries.

**Table 5:** ROC Analysis Results for Differentiation of MINOCA and Non-Critical Coronary Artery Disease

Variable	Cut-off	AUC	Sensitivity	Specificity	PPV	NPV	P value
Neutrophil	≥ 5.4	0.665	0.522	0.749	0.402	0.829	< 0.001
SII	≥ 534.29	0.630	0.582	0.667	0.361	0.831	0.002
NLR	≥ 2.19	0.634	0.627	0.618	0.347	0.837	0.001
LVEF	≤ 55	0.697	0.567	0.792	0.469	0.850	< 0.001

AUC: area under the curve; PPV: positive predictive value; NPV: negative predictive value; SII: systemic immune-inflammation index; NLR: neutrophil-to-lymphocyte ratio; LVEF: left ventricular ejection fraction; ROC: receiver operating characteristic; MINOCA: myocardial infarction with non-obstructive coronary arteries.

## Discussion

MINOCA represents a heterogeneous clinical condition with multiple underlying mechanisms including plaque disruption, coronary vasospasm, microvascular dysfunction, myocarditis, and stress-induced cardiomyopathy [7, 13].

Previous studies have reported that MINOCA accounts for approximately 5–10% of all myocardial infarction cases and differs from obstructive myocardial infarction in important demographic and clinical characteristics [14, 15]. The heterogeneous pathophysiological mechanisms underlying MINOCA have been increasingly recognized, highlighting the importance of identifying reliable biomarkers that may assist in differentiating MINOCA from obstructive myocardial infarction in clinical practice [7, 16].

The main findings of the present study were as follows: systemic inflammatory markers, including neutrophil count, SII and NLR, differed significantly among the three study groups. While inflammatory marker levels were highest in the obstructive myocardial infarction group, intermediate levels were observed in the MINOCA group and the lowest levels in the non-critical coronary artery disease group. In addition, LVEF and SII were identified as independent predictors of MINOCA in multivariate logistic regression analyses. These findings suggest that systemic inflammatory parameters may contribute to the diagnostic evaluation and risk stratification of patients with suspected MINOCA.

Coronary artery disease is known to predominantly affect older individuals, and increasing age is an established risk factor for obstructive myocardial infarction [17]. In the present study, patients in the obstructive myocardial infarction group were significantly older than those in the MINOCA and non-critical coronary artery disease groups, which is consistent with previous reports in the literature. Similarly, male sex was significantly more frequent in the obstructive myocardial infarction group. This finding may be explained by the protective effects of estrogen against the development of obstructive coronary artery disease in women and supports previous studies demonstrating a higher prevalence of MINOCA among relatively younger and female patients compared with obstructive myocardial infarction [18]. In the present study, patients with obstructive myocardial infarction were older and more frequently male compared with patients with MINOCA, which is consistent with previously published epidemiological observations [19].

Traditional cardiovascular risk factors such as diabetes mellitus and hypertension play a major role in the development of obstructive coronary artery disease. In the present study, both diabetes mellitus and hypertension were significantly more prevalent in the obstructive myocardial infarction group than in the MINOCA and non-critical coronary artery disease groups, which is consistent with previously published data [19, 20]. These findings support the concept that classical atherosclerotic risk factors are more strongly associated with obstructive coronary artery disease, whereas MINOCA may involve alternative underlying mechanisms. In contrast, no statistically significant difference was observed among the groups in terms of smoking status. This finding may reflect the multifactorial nature of MINOCA pathophysiology and the relatively high prevalence of smoking in the general population.

Laboratory findings in our study also demonstrated significant differences among the study groups. Serum glucose, creatinine, LDL cholesterol, and white blood cell levels were significantly higher in the obstructive myocardial infarction group compared with the MINOCA and non-critical coronary artery disease groups. These findings are consistent with the established association between metabolic burden, renal dysfunction, and systemic inflammatory activation in patients with obstructive coronary artery disease. In addition, serum albumin levels were significantly lower in the obstructive myocardial infarction group, supporting the role of hypoalbuminemia as a marker of systemic inflammation and adverse cardiovascular outcomes. Taken together, these results further support the concept that patients with obstructive myocardial infarction have a higher inflammatory and metabolic risk profile compared with patients with MINOCA and non-critical coronary artery disease.

Systemic inflammatory indices derived from routine hematological parameters have recently gained attention as potential markers reflecting inflammatory burden in cardiovascular diseases [21]. In the present study, inflammatory indices including neutrophil count, SII, NLR, PLR, MLR, CRP, and CAR were significantly higher in the obstructive myocardial infarction group compared with the MINOCA and non-critical coronary artery disease groups. Notably, patients with MINOCA demonstrated intermediate levels of these inflammatory markers, suggesting that MINOCA represents a distinct clinical entity with a lower inflammatory burden than obstructive myocardial infarction but higher inflammatory activity than non-critical coronary artery disease. Systemic inflammatory indices including NLR, PLR, and SII were significantly higher in the obstructive myocardial infarction group compared with the MINOCA group. These findings are consistent with previous studies demonstrating the association between elevated inflammatory indices and plaque instability, thrombus burden, and adverse cardiovascular outcomes in acute coronary syndromes [22].

Although SII and NLR were significantly associated with MINOCA in univariate analyses, their independent associations differed according to the comparison group. In the comparison between MINOCA and obstructive myocardial infarction, neither SII nor NLR remained independently associated with MINOCA after multivariable adjustment, whereas SII remained an independent predictor when MINOCA was compared with non-critical coronary artery disease. These findings suggest that systemic inflammatory indices may provide supportive, context-dependent diagnostic information rather than serving as standalone predictors in the differentiation of MINOCA. To the best of our knowledge, limited data exist in the literature comparing multiple systemic inflammatory indices simultaneously among MINOCA, obstructive myocardial infarction, and non-critical coronary artery disease groups. Therefore, our findings contribute to the growing body of evidence regarding the role of inflammatory activation in the pathophysiology of MINOCA. Consistent with these findings, CRP and CAR levels were significantly higher in patients with obstructive myocardial infarction, further supporting the role of systemic inflammation in the pathophysiology of plaque rupture and coronary thrombosis. Recent meta-analyses have further demonstrated the prognostic and diagnostic importance of systemic inflammatory indices in patients with acute coronary syndromes [23].

Left ventricular systolic function is an important indicator of myocardial injury severity in patients presenting with acute coronary syndromes [20]. In the present study, LVEF values were significantly lower in the obstructive myocardial infarction group compared with the MINOCA and non-critical coronary artery disease groups. Furthermore, LVEF was identified as an independent predictor of MINOCA in multivariate logistic regression analysis when compared with obstructive myocardial infarction. This finding suggests that patients with MINOCA tend to have relatively preserved left ventricular systolic function compared with patients with obstructive myocardial infarction, which is consistent with the concept that myocardial injury is generally less extensive in MINOCA [24]. These results support the clinical value of echocardiographic evaluation in the differential diagnosis of MINOCA and obstructive myocardial infarction. Lower LVEF values observed in patients with obstructive myocardial infarction in our study are also in agreement with previous reports showing more pronounced myocardial injury and worse ventricular function in obstructive coronary artery disease compared with MINOCA patients [25].

In addition to conventional echocardiographic parameters, advanced echocardiographic imaging techniques, particularly speckle-tracking echocardiography (STE), may provide complementary information in patients with MINOCA. Although LVEF demonstrated the highest diagnostic performance among the parameters evaluated in the present study, myocardial strain analysis may detect subtle myocardial dysfunction despite preserved LVEF. Previous evidence indicates that global longitudinal strain can identify subclinical myocardial impairment in patients with MINOCA and may improve diagnostic characterization beyond conventional systolic function assessment [26]. Furthermore, STE-derived deformation parameters have been associated with myocardial fibrosis and tissue remodeling, supporting their potential role as noninvasive markers when cardiac magnetic resonance imaging is not immediately available [27]. Integrating systemic inflammatory indices with myocardial strain assessment may therefore improve the identification of occult myocardial injury and refine diagnostic evaluation and risk stratification in patients with MINOCA. However, strain parameters were not available in the present retrospective dataset, and their incremental value could not be directly evaluated.

ROC curve analyses were performed to evaluate the diagnostic performance of inflammatory parameters and echocardiographic findings in distinguishing MINOCA from obstructive myocardial infarction. Among the evaluated parameters, LVEF demonstrated the highest diagnostic performance, followed by neutrophil count, NLR, and SII. Although the sensitivity values of these parameters were moderate, their relatively higher specificity suggests that these parameters may be useful as supportive tools in the differential diagnosis of MINOCA in clinical practice. In particular, the identification of practical cut-off values for LVEF, neutrophil count, NLR, and SII may facilitate early risk stratification and diagnostic evaluation in patients presenting with suspected myocardial infarction without significant coronary artery obstruction. ROC curve analysis demonstrated that neutrophil count, SII, NLR, and LVEF had moderate diagnostic performance in distinguishing MINOCA from obstructive myocardial infarction. Similar findings have been reported in previous studies evaluating the diagnostic utility of systemic inflammatory indices in acute coronary syndromes. Previous studies have emphasized the diagnostic challenges associated with MINOCA and suggested that additional laboratory and imaging markers may contribute to improved differentiation between MINOCA and obstructive myocardial infarction [28].

Notably, patients with MINOCA exhibited an intermediate inflammatory profile, with inflammatory marker levels higher than those observed in patients with non-critical coronary artery disease but lower than those observed in patients with obstructive myocardial infarction. This finding supports the concept that MINOCA represents a distinct pathophysiological entity characterized by a relatively milder degree of systemic inflammation.

Lifestyle modification may also represent an important component of the comprehensive management of patients with MINOCA. Although the present study focused on inflammatory indices and echocardiographic parameters rather than therapeutic interventions, lifestyle-related factors may influence systemic inflammation, endothelial function, symptom burden, and overall cardiovascular risk. Recent evidence in patients with angina and non-obstructive coronary arteries suggests that a structured multidomain intervention incorporating exercise training, dietary counseling, psychological support, and intensive risk-factor management may improve patient-reported health status when added to mechanism-directed medical therapy [29, 30]. Accordingly, smoking cessation, regular physical activity, dietary optimization, weight management, blood pressure control, and glycemic control should be considered as part of individualized secondary prevention strategies in patients with MINOCA.

MINOCA represents a heterogeneous clinical condition with complex underlying mechanisms, and its diagnostic evaluation remains challenging in routine clinical practice. The identification of easily accessible and cost-effective laboratory markers that may assist in the differentiation of MINOCA from obstructive myocardial infarction and non-critical coronary artery disease is therefore of considerable clinical importance. In this context, our study demonstrated that systemic inflammatory indices, together with echocardiographic parameters such as LVEF, may provide additional value in the evaluation of patients with suspected MINOCA. Moreover, the simultaneous comparison of multiple inflammatory indices across three clinically relevant patient groups represents an important strength of the present study and contributes to the limited existing literature on the role of inflammatory activation in MINOCA.

Future prospective multicenter studies incorporating routine cardiac magnetic resonance and intracoronary imaging are needed to further clarify the prognostic role of systemic inflammatory indices in patients with MINOCA.

### Limitations

The present study has several limitations that should be considered when interpreting the results. First, advanced imaging modalities such as cardiac magnetic resonance imaging and intracoronary imaging techniques were not routinely performed in all patients, which may have limited accurate identification of the underlying mechanisms of MINOCA. Second, this was a retrospective single-center study, which may restrict the generalizability of the findings to broader patient populations. Third, the inclusion of an elective angiography group may have introduced potential selection bias. Fourth, STE-derived strain parameters were not available, so their incremental diagnostic value could not be evaluated. Finally, long-term clinical follow-up data were not available; therefore, the prognostic implications of the evaluated inflammatory parameters could not be assessed.

### Conclusions

Systemic inflammatory indices, particularly SII, together with LVEF, may serve as easily accessible supportive markers in differentiating MINOCA from obstructive myocardial infarction and non-critical coronary artery disease. However, their moderate supportive performance suggests that they should be used in combination with multimodality imaging rather than as standalone diagnostic tools.

Further prospective multicenter studies with comprehensive imaging evaluation are needed to clarify the role of systemic inflammatory indices in the supportive assessment of patients with MINOCA.

## Data Availability

The datasets generated and analyzed during the current study are available from the corresponding author on reasonable request.
